# Severe acute respiratory syndrome coronavirus-2 (SARS-CoV-2) and coronavirus disease 19 (COVID-19) – anatomic pathology perspective on current knowledge

**DOI:** 10.1186/s13000-020-01017-8

**Published:** 2020-08-14

**Authors:** Sambit K. Mohanty, Abhishek Satapathy, Machita M. Naidu, Sanjay Mukhopadhyay, Shivani Sharma, Lisa M. Barton, Edana Stroberg, Eric J. Duval, Dinesh Pradhan, Alexandar Tzankov, Anil V. Parwani

**Affiliations:** 1 Department of Pathology and Laboratory Medicine, CORE Diagnostics, Gurgaon, India; 2 Department of Pathology and Laboratory Medicine, Advanced Medical Research Institute and Prolife Diagnostics, Bhubaneswar, India; 3 Department of Pathology, Cleveland Clinic, Cleveland, OH USA; 4 Office of the Chief Medical Examiner, Oklahoma City, OK USA; 5 Aurora Diagnostics, Jacksonville, FL USA; 6 Institute of Medical Genetics and Pathology, University Hospital Basel, Baseland, Liestal, Switzerland; 7 Department of Pathology, The Ohio State University, E409 Doan Hall, 410 West 10th Ave, Columbus, OH 43210 USA

**Keywords:** COVID-19, Autopsy, Pathology, Pathogenesis

## Abstract

**Background:**

The world is currently witnessing a major devastating pandemic of Coronavirus disease-2019 (COVID-19). This disease is caused by a novel coronavirus named Severe Acute Respiratory Syndrome Coronavirus-2 (SARS-CoV-2). It primarily affects the respiratory tract and particularly the lungs. The virus enters the cell by attaching its spike-like surface projections to the angiotensin-converting enzyme-2 (ACE-2) expressed in various tissues. Though the majority of symptomatic patients have mild flu-like symptoms, a significant minority develop severe lung injury with acute respiratory distress syndrome (ARDS), leading to considerable morbidity and mortality. Elderly patients with previous cardiovascular comorbidities are particularly susceptible to severe clinical manifestations.

**Body:**

Currently, our limited knowledge of the pathologic findings is based on post-mortem biopsies, a few limited autopsies, and very few complete autopsies. From these reports, we know that the virus can be found in various organs but the most striking tissue damage involves the lungs resulting almost always in diffuse alveolar damage with interstitial edema, capillary congestion, and occasional interstitial lymphocytosis, causing hypoxia, multiorgan failure, and death. A few pathology studies have also reported intravascular microthrombi and pulmonary thrombembolism. Although the clinical presentation of this disease is fairly well characterized, knowledge of the pathologic aspects remains comparatively limited.

**Conclusion:**

In this review, we discuss clinical, pathologic, and genomic features of COVID-19, review current hypotheses regarding the pathogenesis, and briefly discuss the clinical characteristics. We also compare the salient features of COVID-19 with other coronavirus-related illnesses that have posed significant public health issues in the past, including SARS and the Middle East Respiratory Syndrome (MERS).

## Background

Coronavirus disease 2019 (COVID-19) is a recent global public health catastrophe with substantial mortality and morbidity across the globe. From its origin in the Hubei province of China in late 2019 (December, 2019), COVID-19 has spread to many countries across the globe and is now a pandemic [1]. The virus causing COVID-19 is a novel beta coronavirus popularly known as Severe Acute Respiratory Syndrome Coronavirus-2 (SARS-CoV-2) [2]. COVID-19 is clinically characterized by high rates of transmission, mild to moderate phenotypic clinical manifestations, and significant clinical, radiologic, and pathologic abnormalities in the elderly [3]. This disease primarily affects the respiratory tract, although efforts are currently ongoing to study its possible effects in other tissues.

## The causative agent: severe acute respiratory syndrome coronavirus-2

**Fig. 1:**
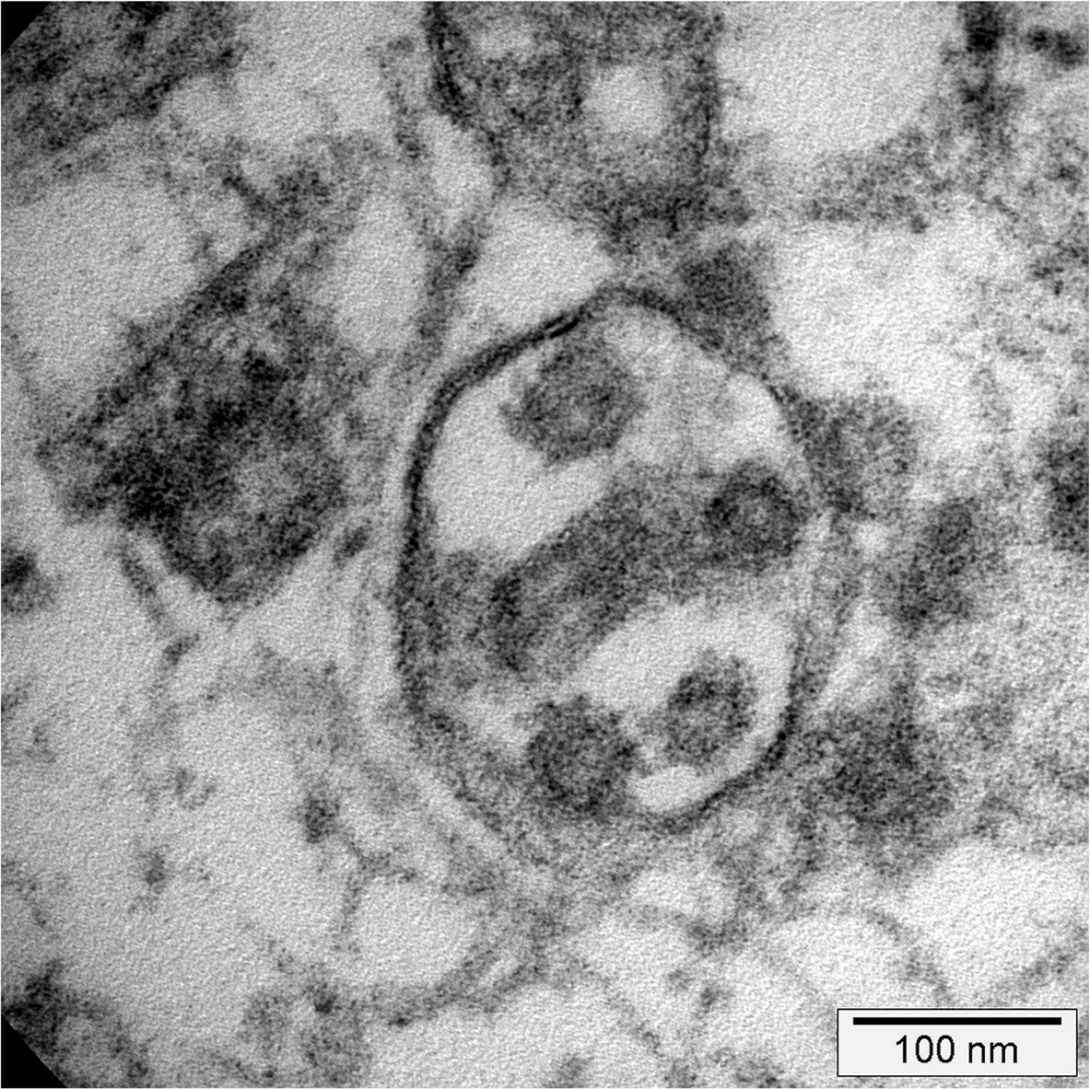
Electron micrograph of the intracellular virus-like particles of a COVID-19 victim (“Courtesy of Martin Herzig for technical preparation and performing the microphotography”)

Coronaviruses are a large, diverse group of enveloped viruses containing positive-sense single-stranded RNA as their genetic material. These viruses are responsible for various respiratory diseases (including the common cold) in humans and other mammals [4]. Coronaviruses are characterized by club-shaped protein spikes on their envelope, giving them a crown-like appearance when viewed by transmission electron microscopy (hence the term Coronavirus) [5]. The virus measures 120 nm in diameter (Fig. 1). Other members of the coronavirus family have also caused major respiratory illnesses. These include SARS-CoV (SARS-CoV-1) the causative agent of the Severe Acute Respiratory Syndrome (SARS), and Middle East Respiratory Syndrome-Related Coronavirus (MERS-CoV), the causative agent of Middle East Respiratory Syndrome (MERS) [6] (Table 1).

**Table 1:** Comparison between corona virus-related illnesses those posed significant public health issues

Characteristics	MERS	SARS	COVID-19
**Status**	**First reported in Saudi Arabia in September 2012, 27 countries affected, 2519 people infected, 866 deaths**	**First reported in Asia in February 2003, 26 countries affected, 8000 people infected, 774 deaths**	**First reported in Wuhan, China in December 2019, causative agent identified in January 2020. 4,525,497 as of 17th May, 2020, with 307,395 deaths and a total of 215 countries/geographic location**
**Causative virus**	**MERS-CoV**	**SARS-CoV**	**SARS-CoV-2**
**Tissue tropism**	**Pneumocytes, pulmonary macrophages, macrophages infiltrating the skeletal muscles, and renal proximal tubular epithelial cells**	**Pneumocytes, lymphocytes, monocytes, and lymphoid tissues, intestinal mucosa, renal distal tubular epithelial cells, neurons in the brain, and tissue-resident macrophages in different organs**	**Pneumocytes, intestinal mucosa, renal distal tubular epithelial cells, and endothelium**
**Clinical Features**	**Severe acute respiratory illness, including fever, cough, and shortness of breath**	**Flu-like illness, including fever, chills, cough, and malaise. 70% of the patients subsequently suffer from shortness of breath and recurrent or persistent fever, and 30% show clinical improvement after the first week**	**Fever, dry cough, fatigue, shortness of breath, bone pain, and sore throat as well as a rather specific smell- and taste sensational loss. Some patients present with gastrointestinal symptoms such as nausea, vomiting, and diarrhea. Some patients have a relatively stable clinical course for 5 to 8 days followed by acute and very rapid deterioration in patients, who may run into a critical course afterwards. Advanced cases generally had respiratory, cardiovascular, and renal failure.**
**Macroscopy/Gross**	**Edematous lungs with increased gross weight and multiple areas of congestion**	**Edematous lungs with increased gross weight and multiple areas of congestion, enlargement of lymph nodes in the pulmonary hila and the abdominal cavity, diminished spleen size, and weight**	**Edematous lungs with increased gross weights, multiple areas of congestion, and pulmonary embolism**
**Microscopy**^**a**^	**Exudative diffuse alveolar damage with hyaline membranes, pulmonary edema, type II pneumocyte hyperplasia, interstitial lymphocytosis, multinucleate syncytial cells, bronchial submucosal gland necrosis, acute tubulointerstitial nephritis, and acute tubular sclerosis with proteinaceous cast formation**	**Bronchial epithelial denudation, loss of cilia, squamous metaplasia, acute diffuse alveolar damage, and in the late phase acute fibrinous and organizing pneumonia**	**Diffuse alveolar damage, severe capillary congestion, interstitial mononuclear cell infiltrates, and multinucleated syncytial cells with atypical enlarged pneumocytes, and occasionally microthrombosis**^**a**^
**Pathogenesis**^**b**^	**Bronchial lesions are pathologic basis for the respiratory failure; DPP4, the entry receptor widely expressed (epithelial cells in the kidney, alveoli, small intestine, liver, prostate, activated leukocytes); robust and sustained production of proinflammatory cytokines; infects and evades the T cell response; induce apoptosis of both kidney and lung cells through upregulation of Smad7 and FGF2**	**Combination of direct virus-induced cytopathic effects and immunopathology induced by a hypercytokinemia or a “cytokine-storm”**	**Combination of direct virus-induced cytopathic effects, immunologic injury, and microvascular damage induced by cytokines**

^a^None of these changes have been shown to be pathognomonic for MERS, SARS, or COVID-19

^b^All statements regarding pathogenesis are hypothetical, albeit with some indirect evidence

Phylogenetically, SARS-CoV-2 shares a major (79%) identity in its nucleotide sequence with SARS-CoV, which caused a major epidemic in 2002–2003 that resulted in 774 deaths in approximately 8000 affected individuals from 26 countries [7]. It is certain that SARS-CoV-2 displays identical sequences for its envelope and nucleocapsid proteins with SARS-CoV in 96 and 89.6%, respectively [7]. MERS-CoV shares 50% homology with SARS-CoV-2. SARS-CoV and SARS-CoV-2 utilize the angiotension-converting enzyme (ACE)-2 as a receptor for their entry into human cells, whereas MERS-CoV utilizes dipeptidyl peptidase (DPP)-4 for cellular entry [7].

SARS-CoV-2 viral genomic decoding was first performed using a metagenomic RNA-sequencing high-throughput platform [8]. To date, over 12,000 samples have been sequenced and shared via the Global Initiative on Sharing All Influenza Data (GISAID) and GenBank, enabling researchers to access sequencing data of the virus [9–11]. The reference genome isolated from Wuhan has the NCBI reference sequence number NC_045512.2. Some strains have revealed progressive mutations, such as EPI_ISL_412973 (Italy), EPI_ISL_408008 (USA), EPI ISL_408430 (France), EPI_ISL_408665 (Japan), etc. [10]. Researchers in China have identified over 100 strains using Illumina and Oxford Nanopore methods. We can now construct a phylogenetic network of viral evolution by using these vast datasets. According to this phylogenetic tree, there are three core variants, with minor variations in their amino acid sequences. The most primitive (“ancestral”) variant is designated ‘A’. Two newer variants are designated B and C, respectively. Geographically, A and C are prevalent outside East Asia, whereas B has a predominant presence in East Asia. The phylogenetic tree serves as a snapshot of the path of the pandemic spread. Mutational variants can be studied individually to understand their clinical behavior and spread. Accurate prediction of epidemiological and clinical outcomes may aid in the formulation of solid preventive strategies. Phylogenetic analysis of SARS-CoV-2 has revealed its tendency to form clusters. They appear to be conserved without branching during current pandemic. Ancestral forms are still in circulation without any major changes [11]. Gene arrangement in the viral genome has been determined through sequencing. Two untranslated regions (UTR) flank the coding region at both 5′ and 3′ ends. Genes in the coding region, from 5′ end to 3′ end are Open Read Frame ab (ORF1ab), spike (S), envelope (E), membrane (M), and nucleocapsid (N). Several other ORFs are also present between S and N genes. ORF1ab is the largest of the genes and is further subdivided into ORF1a and ORF1b. The ORF1ab gene encodes more than 15 nonstructural proteins including RNA-dependent RNA polymerase (RdRP) and helicase [12].

## Commencement of the global disaster

In December 2019, many adults in Wuhan, the capital city of Hubei province of China, presented to local hospitals with severe respiratory disease of unknown cause. Many of the initial cases had a common exposure to the Huanan wholesale seafood market where live animal trading occurred. Eventually, people with no link with the seafood market also presented in similar fashion pointing towards human to human transmission. China notified the World Health Organization (WHO) of the outbreak on 31st December 2019 and on 1st January 2020, following which the Huanan seafood market was shut down. On 7th December 2019, the causative agent was identified as a coronavirus. Even though the origin of SARS-CoV-2 has not been determined with certainty, it may believed to have originated from bats, possibly involving civets or pangolins as intermediates, and possibly originated from Wuhan [7]. This hypothesis is based on the observations that these mammals harbor a high diversity of coronaviruses, and SARS-CoV-2 has 96 and 99% genetic homology with coronaviruses found in bats and pangolin species, respectively.

Interestingly, the Wuhan Institute of Virology is a leading center of research involving coronaviruses, and published a paper in *Science* back in 2018 entitled “Bats are natural reservoirs of SARS-like coronaviruses” [13]. As of the time of this writing (May, 20, 2020), we are not aware of any peer-reviewed publications definitively linking the research performed in this laboratory to the COVID-19 outbreak.

## Epidemiology of COVID-19

According to the WHO, the latest number of COVID-19 positive cases is 4,993,470 as of 22nd May, 2020, with 327,758 deaths and a total of 216 countries/geographic locations involved (https://covid19.who.int). The cases are occurring in clusters and are gradually developing into widespread outbreaks [14]. The majority of positive cases have affected individuals aged between 30 years and 85 years, and over half have been reported in males [15]. About half of all patients have had associated comorbidities such as hypertension, diabetes, cardiovascular disease, and so on [16]. Case fatality is significantly increased in the presence of comorbidities [15, 16]. Interestingly, despite the fact that HIV-positive individuals with advanced disease, high viral load, and low CD4 count, in general have a high susceptibility of acquiring new infection and related complications, according to the WHO, it is unknown whether HIV seropositivity or AIDS-associated immunosuppression have any role in COVID-19 prevalence and predisposition. Yet, it is sensible for immunosuppressed individuals to take additional precautions.

## Route(s) of transmission

The principal mode of transmission is through the respiratory route, primarily by large droplets or aerosols. Infected surfaces and fomites have been the routes of infection in some instances [17]. Typically, close and prolonged contact for over 15 min significantly increases the likelihood of contracting the infection. Infectivity is greatly increased by repeated or prolonged exposure, making healthcare workers particularly vulnerable. Spread and impact are determined by the R0 and case fatality rate values. R0 value is the number of secondary infections from a case; values over 1 imply a proclivity for spread. During the early spread of COVID-19, the R0 value was between 2.2 and 3.58 [18]. These values vary greatly for COVID-19, primarily because of under-reporting of mild and asymptomatic cases and universal non-uniformity of testing facilities. COVID-19 is more widespread and less lethal than SARS and MERS. The mean incubation period has been 5 days so far. However, some reported cases have shown an unusually prolonged incubation period - as high as 24 days [19]. Viral shedding occurs during the convalescent period (range from 8 days to 37 days, with a median of 20 days). However, in fatal cases, it continues till death.

## Pathogenesis

**Fig. 2:**
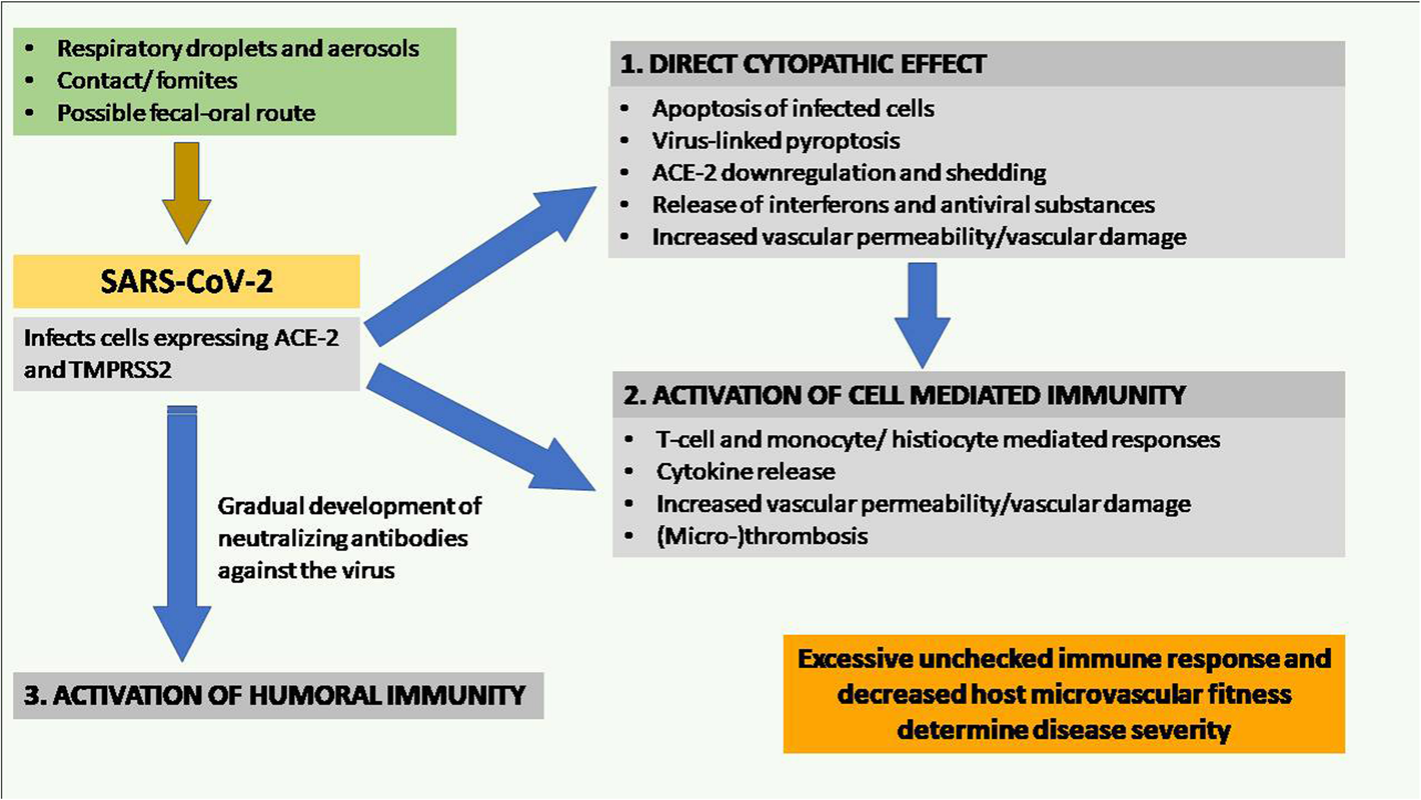
Pathogenesis of COVID-19

Envelope-based spike protein (S protein) is the principal determinant of virulence [20]. This protein determines the specific tissue affinity or tissue tropism, infectivity, and species diversity. The S1 domain of this protein is responsible for receptor binding, whereas the S2 domain is crucial for cell membrane fusion [21]. The S protein is cleaved at the S2 site, present just adjacent to fusion peptide by host protease TMPRSS2. This event causes permanent structural change to facilitate viral entry into susceptible cells [22]. For SARS-CoV and SARS-CoV-2, the receptor is ACE-2 [23]. SARS-CoV-2 has a higher affinity for the receptor (10 to 20 times greater than SARS-CoV), favoring rapid spread among the human population [24]. Importantly, there is evidence from SARS-CoV that blood group antigen A may directly interact with the viral S protein [25], thus facilitating virus entry via ACE-2, which was postulated to have a direct effect on the number of infected individuals and disease kinetics in SARS, but may also explain the higher incidence of blood group A among COVID-19 patients reported in a large populational study from China [26] and corroborated by the largest autopsy series thus far [27]. ACE-2 has a wide species distribution facilitating cross-species transmission. Interestingly, ACE-2 are also reported to be expressed in the kidney and gastrointestinal tract in addition to the respiratory system [22, 23, 28] (Fig. 2). A recent report suggests that SARS-CoV-2 RNA can be detected in the fecal matter of some patients of COVID-19 [29]. This, along with the fact that some patients with this disease also have diarrhea, points to the possibility of the involvement of the gut-lung axis and raises the possibility that the gastrointestinal system may be another portal of entry and/or a site of disease [30]. Also, diarrhea secondary to mucosal ischemia following microvascular damage may be an attributable factor.

## Clinical features

The clinical spectrum of COVID-19 disease ranges from mild flu-like symptoms to severe pulmonary damage with acute respiratory distress syndrome (ARDS). There are a large number of undiagnosed, asymptomatic, and subclinical cases [31]. According to the WHO, 5% of the cases are critical, 15% are severe, 40% are moderate, and 40% are mild (https://www.who.int/docs/default-source/coronaviruse/risk-comms-updates/update-18-epi-win%2D%2Dcovid-19.pdf?sfvrsn=cfb0471f_2). Meta-analyses of hospitalized patients have revealed that 18% of the hospitalized patients had severe disease, with 15% having ARDS [32]. The clinical symptoms during the initial presentation included fever (88%), dry cough (68%), fatigue (38%), shortness of breath (19%), bone pain (15%), and sore throat (https://www.who.int/docs/default-source/coronaviruse/who-china-joint-mission-on-covid-19-final-report.pdf) as well as a rather specific smell- and taste sensational loss [33]. Some patients also had gastrointestinal symptoms such as nausea, vomiting, and diarrhea. Some patients have a relatively stable clinical course for 5 to 8 days followed by acute and very rapid deterioration in patients, who may run into a critical course afterwards. Advanced cases generally had respiratory, cardiovascular, and renal failure. Ultimately, death, in most instances, was due to multiorgan failure [34].

## Imaging features

**Fig. 3:**
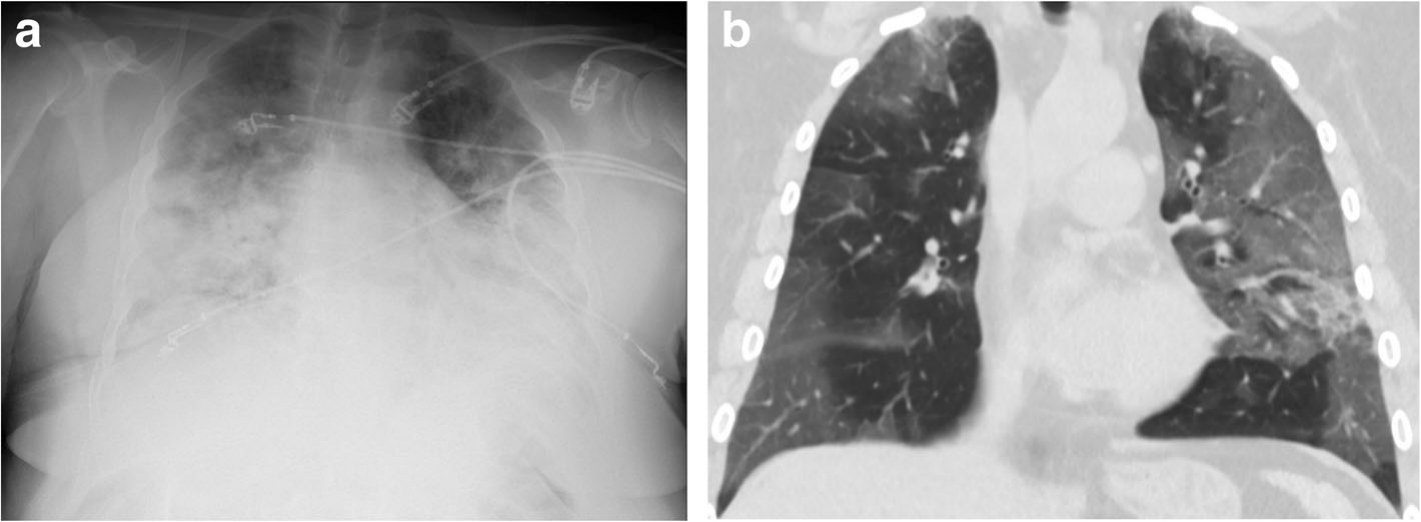
**a** and **b** Imaging in COVID-19 showing widespread bilateral interstitial and airspace opacities (3B: “Courtesy of Dr. Maurice Henkel”)

The range of reported imaging features of COVID-19 includes bilateral patchy ground-glass opacities, extensive bilateral interstitial, and air space opacities (Fig. 3a and b), and consolidation with air bronchograms [35, 36]. Yet, normal CT scans have been reported in some patients.

In the most severe cases, complete “white-out” has been noted. Interestingly, abnormalities on chest imaging have also been noted in asymptomatic carriers [37]. The distribution of the opacities is usually peripheral. Accompanying ground-glass opacities and fine reticular opacities were also identified. Central distribution of infiltrates, pleural involvement with effusion, and lymphadenopathy were less frequent.

## Pathologic features of COVID-19

There is currently a paucity of pathologic data on COVID-19, most of it being derived from observations reported in a few post-mortem biopsies, two lung cancer lobectomies, a handful of limited autopsies and only a few complete autopsies, including 2 recent series [27, 38–48]. Published reports are summarized in Table 2. Although autopsy data are limited, overall the pathologic findings reported thus far are similar to those reported in H1N1 (swine flu) [60] and SARS [61].

**Table 2:** Histopathology of COVID-19

Authors	PMID	Specimen type	No. of cases	Main findings	DAD	Thrombi
Xu et al. [38]	32,085,846	Post-mortem biopsies of lung, liver, and heart	1	DAD	Yes	None mentioned
Tian et al. [39]	32,114,094	Lobectomies	2	DADand mononuclear inflammatory cells	Yes (early DAD pattern in 1 of 2)	None mentioned
Barton et al. [40]	32,275,742	Complete autopsies	2	DAD and chronic airway inflammation	Yes (1 case)	Few (lung, case 1)
Karami et al. [41]	32,283,217	Autopsy of the lungs	1	Hyaline membranes and viral cytopathic effect	Yes (hyaline membrane noted)	None mentioned
Tian et al. [42]	32,291,399	Post-mortem biopsies of lung, liver, and heart	4	DAD	Yes	None mentioned
Magro et al. [43]	32,299,776	Limited autopsies (2) and skin biopsies (3)	5	“Hemorrhagic pneumonitis” (lung), and “thrombogenic vasculopathy” (skin)	Yes (hyaline membranes in 1 of 2 cases in which lungs were examined)	Yes (skin)
Barnes et al. [44]	32,302,401	Autopsies (brief mention)	3	“Neutrophil extracellular traps”	Not mentioned	None mentioned
Varga et al. [45]	32,325,026	Autopsies (2) and small intestine resection (1)	3	“Endothelitis”, DAD, and viral inclusions in the endothelial cells of kidney	Yes	“Only scattered fibrin thrombi”
Konopka et al. [46]	32,360,729	Autopsy	1	“Fibrinous pneumonia”	Yes	“Rare fibrin thrombi were also identified within small vessels and a small muscular pulmonary artery”
Menter et al. [27]	32,364,264	Autopsy	21	DAD (exudative in 16, proliferative in 8); Superimposed bronchopneumonia in 10/21	Yes	Pulmonary embolism in 4/21; microthrombi of alveolar capillaries in 5/11
Wichmann et al. [47]	32,374,815	Complete autopsies	12	DAD in 8/12; “focal bronchopneumonia” (no DAD) in 4/12	Yes	“Massive pulmonary embolism” (4/12); deep vein thrombosis in 3; fresh thrombosis in prostatic venous plexus (6/9 men)
Lax et al. [48]	32,422,076	Autopsies	11	DAD in 11 /11; bronchopneumonia (6/11); Fibrous adhesions (7/11)	Yes	Thrombosis of small and mid-sized pulmonary arteries (11/11)
Yan et al. [49]	32,422,081	Complete autopsy	1	DAD	Yes	Pulmonary infarction
Buja et al. [50]	32,434,133	Complete autopsies	3	DAD	Yes	Pulmonary embolism in 1/3
Martinez et al. [51]	32,437,316	Complete autopsies	8	DAD	Yes	Fibrinous thrombi in 1/8
Schaller et al. [52]	32,437,497	Complete autopsies	10	DAD	Yes	____
Duarte-Neto et al. [53]	32,443,177	Post-mortem “biopsies”	10	DAD	Yes	Fibrinous thrombi in 8/10; small thrombi in kidneys (glomeruli) and other organs
Sekulic et al. [54]	32,451,533	Complete (1) and partial(1) autopsy	2	DAD	Yes	_______
Aguiar et al. [55]	32,458,044	Complete autopsy	1	DAD, superimposed pneumonia	Yes	_______
Schaefer et al. [56]	32,561,849	Autopsies	7	Acute DAD (2/7), organizing DAD (5/7)	Yes	Pulmonary thromboembolism (5/7)
Beigmohammadi et al. [57]	32,552,178	Post-mortem biopsies from Lung, Heart and Liver	7	DAD (5/7), Acute pneumonia (2/7)	Yes	None mentioned
Konopka et al. [58]	32,542,743	Limited autopsies	8	DAD (8/8)	Yes	Fibrin thrombi (5/8)
Escher et al. [59]	32,529,795	Endomyocardial biopsy	5	Myocardial necrosis, small arterial obliteration	Not applicable	None mentioned

*Abbreviation*: *DAD* Diffuse alveolar damage

### Macroscopic features

**Fig. 4:**
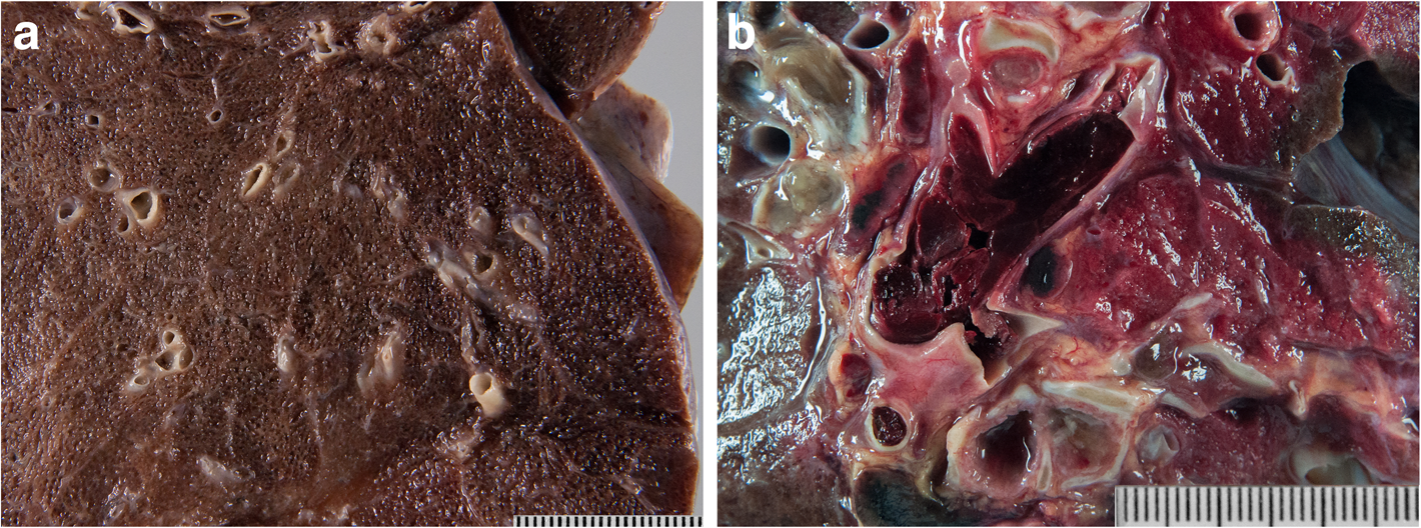
Gross Images of a COVID-19 lung with **a** interstitial edema and congestion; **b** pulmonary embolism in COVID-19

Gross findings in COVID-19 are non-specific. The lungs are heavy, with bilateral interstitial edema and congestion [39, 41, 44]. The cut surfaces show tan-grey consolidation and/or patchy hemorrhagic areas [27]. Wichmann et al. [47] and Menteret al [27]. have reported grossly visible pulmonary emboli (in both studies in 1/3 of patients), and a peculiar patchy gross appearance of the lung parenchyma (both externally as well as on the cut sections), and thrombosis of the prostatic vein (6 of 9 men) [47]. Pleural adhesions were identified in one complete autopsy [40] (Fig. 4a and b).

### Microscopic features

**Fig. 5:**
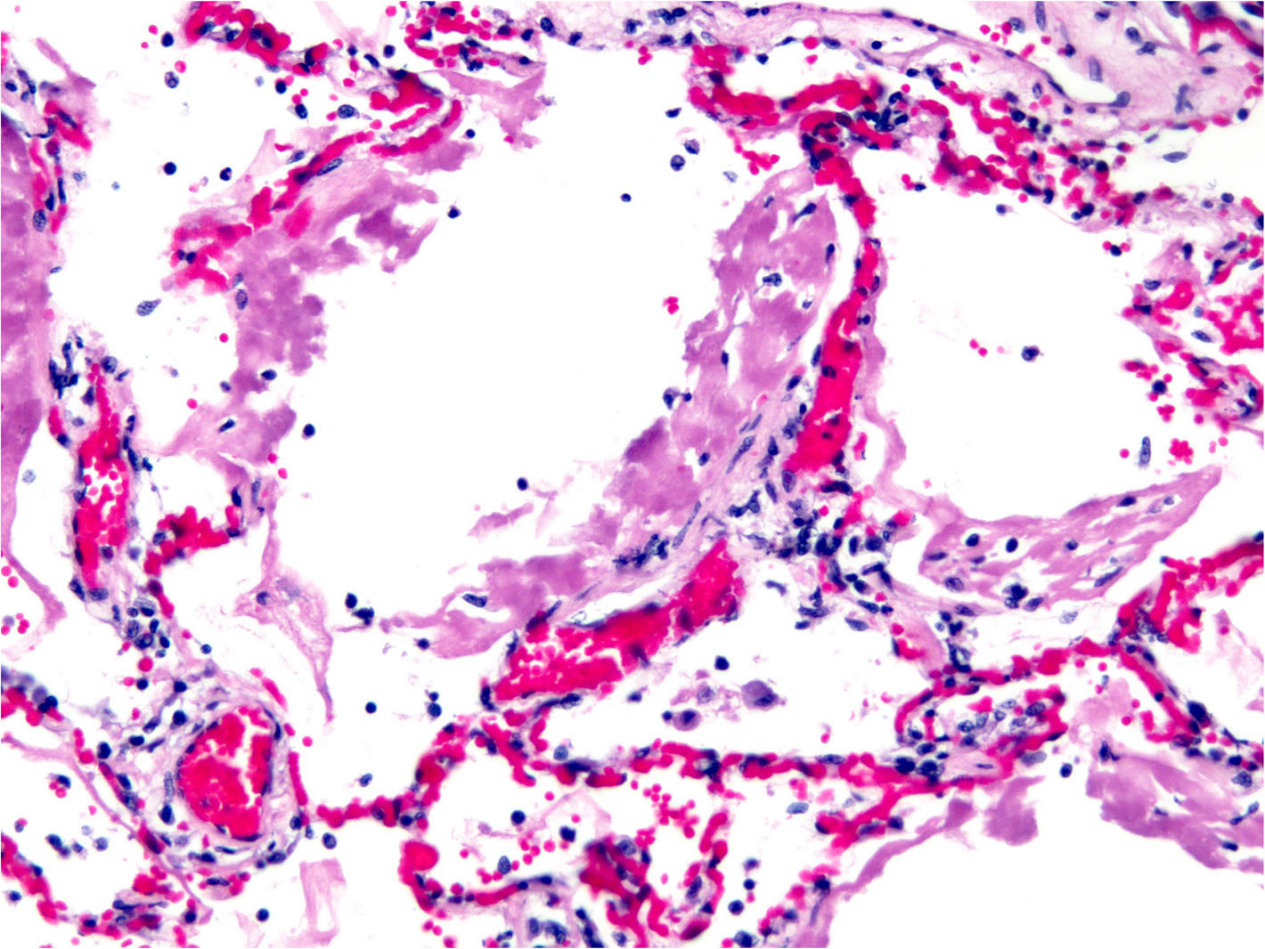
Diffuse alveolar damage in COVID-19. Note prominent hyaline membranes. (Hematoxylin & eosin, original magnification × 200). There is no evidence of “diffuse microthrombi”

Autopsy data have thus far revealed non-specific histopathologic findings, none of which are pathognomonic of COVID-19; pulmonary changes have been the most noteworthy. The most commonly reported pathologic finding in fatal cases thus far has been diffuse alveolar damage (DAD) (Fig. 5 and Table 2). Both acute (exudative) and organizing (fibroproliferative) phases have been reported [27, 41, 42, 44]. In many cases, DAD occurred even in the absence of ventilation, providing evidence that DAD in these patients is caused by viral damage rather than ventilator-induced or oxygen-induced injury [27, 40]. As with DAD in other settings, the acute stage is characterized by the presence of hyaline membranes, and the organizing phase is characterized by variable degrees of proliferation of fibroblasts and myofibroblasts. COVID-19 cannot be differentiated from the other etiologies of DAD by morphologic evaluation. As in other causes of DAD, prominent type 2 pneumocytes have been reported [27]. These cells show cytomegaly, nucleomegaly, and prominent bright eosinophilic nucleoli. These changes are frequently encountered in other infectious and non-infectious causes of DAD [60–62]. No consistent or pathognomonic viral cytopathic effects have yet been established [38, 41]. One study reported remarkable capillary congestion observable in all 21 specimens, irrespective of DAD, accompanying suppurative pneumonia etc. [27].

**Fig. 6:**
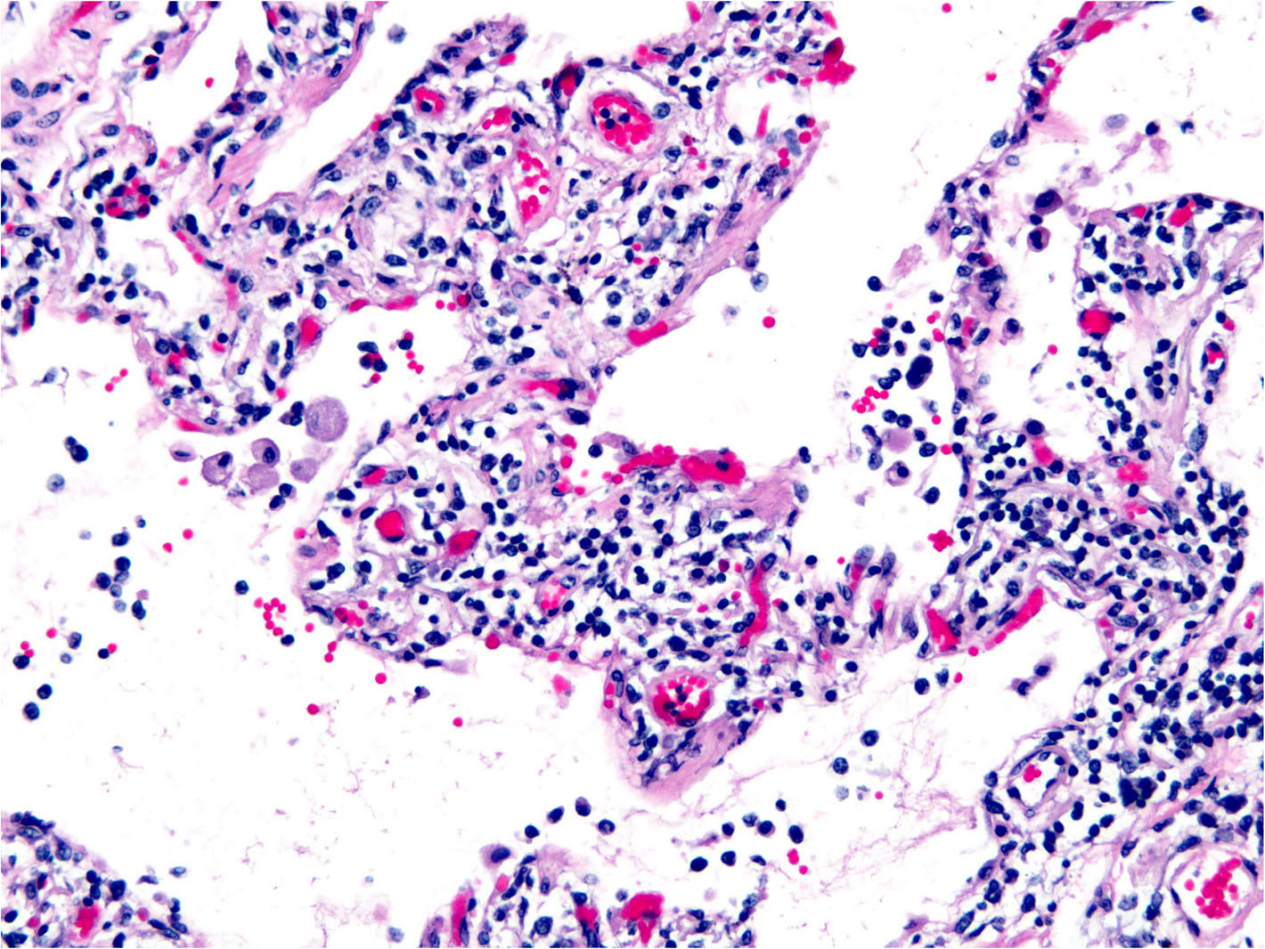
Interstitial inflammation in COVID-19. The inflammatory cells are predominantly lymphocytes (Hematoxylin & eosin, original magnification × 200)

Abnormalities in the interstitium have also been described [27, 40]. The alveolar septa show patchy expansion by a mild to moderate inflammatory infiltrate composed primarily of lymphocytes (Fig. 6). These cells are an admixture of CD4+ and CD8 + T-lymphocytes. However, focal collections of neutrophils have also been reported in a subset of cases. These have been variably interpreted as acute bronchopneumonia [27, 42, 44], “neutrophil extracellular traps”, or, in rare instances, capillaritis [44]. Since neutrophils are not typically encountered in uncomplicated viral infection and do typically occur in superimposed bacterial infection, it is unclear whether the neutrophils reported in COVID-19 cased are related to viral injury or reflect a superimposed bacterial infection, or other unrelated processes [42, 43]. Indeed, aspiration pneumonia (acute bronchopneumonia with neutrophils and food particles) has been reported in one complete autopsy of a COVID-19 decedent, suggesting that these cells arenot a component of the primary inflammatory response in COVID-19 [40].

**Fig. 7:**
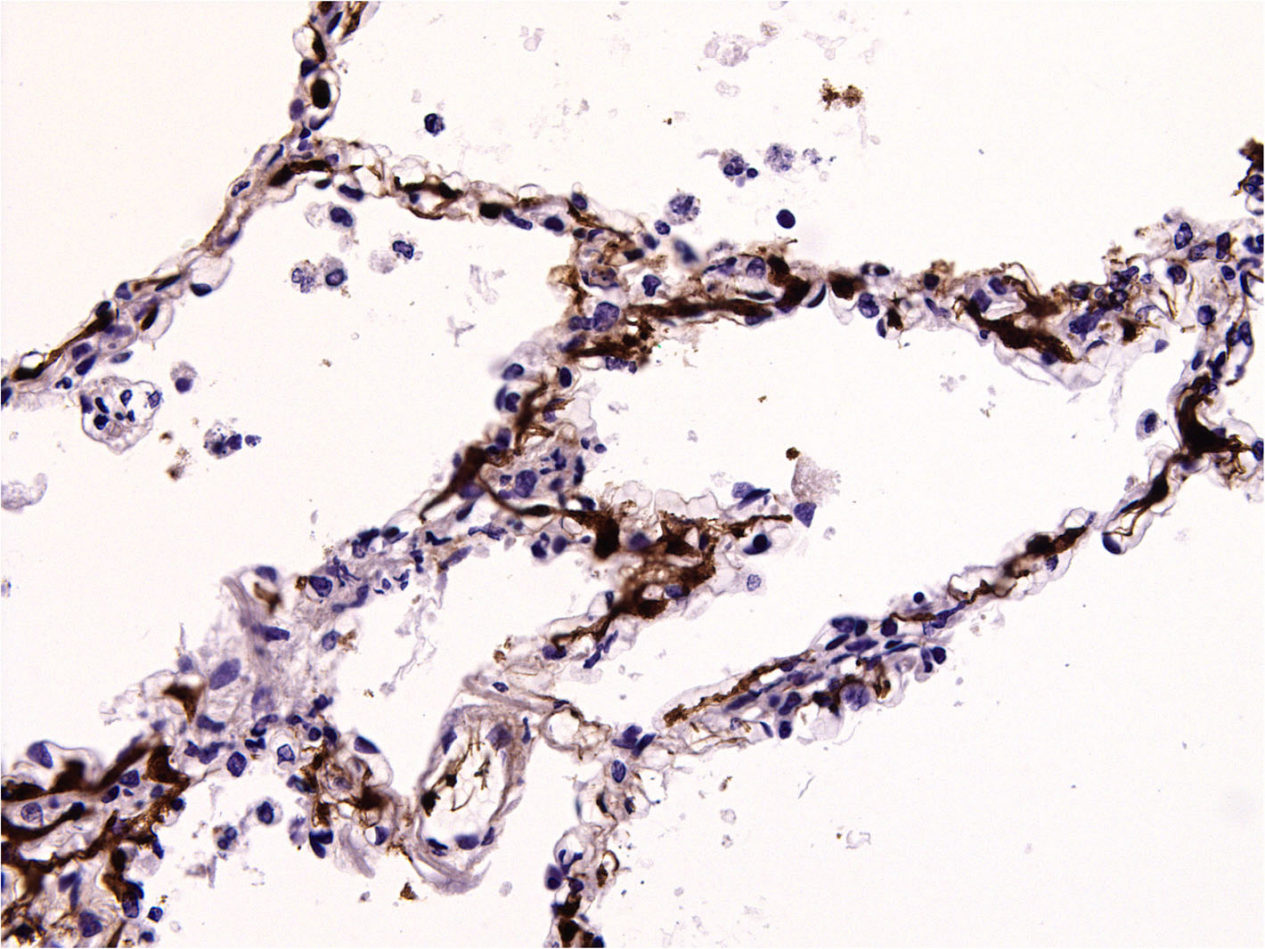
COVID-19 lung with microthombi casting the capillaries of the alveoli (Fibrin stain, original magnification × 200)

There are several clinical reports of thrombosis in COVID-19 patients [63, 64], and the hypothesis of a “pulmonary-specific vasculopathy” has been postulated [65]. However, the pathology literature has only recently provided a few well documented examples of bona fide thrombosis, including a few cases of pulmonary embolism, thrombosis of the prostatic veins, microthrombi in alveolar capillaries [27], and thrombi in glomerular capillaries (3/18 cases in Menteret al.) [27] (Table 2, Fig. 7). One recent report illustrates a few thrombi in skin biopsies from 3 COVID-19 patients who presented with retiform purpura or livedo racemosa [43]; of note, no thrombi are illustrated in any other organ in this report; figure 1b of the manuscript, which illustrates lung sections from a limited autopsy, shows alveolated lung at low magnification precluding accurate assessment of the status of the capillaries.

In summary, it now seems clear that clinical, hematologic, or pathologic evidence of thrombosis is present at least in a subset of COVID-19 cases [64, 66–69]. However, the pathology community must continue to demand that cases claiming significant vascular pathology be adequately illustrated to support the claim of “widespread microthrombosis” in COVID-19.

**Fig. 8:**
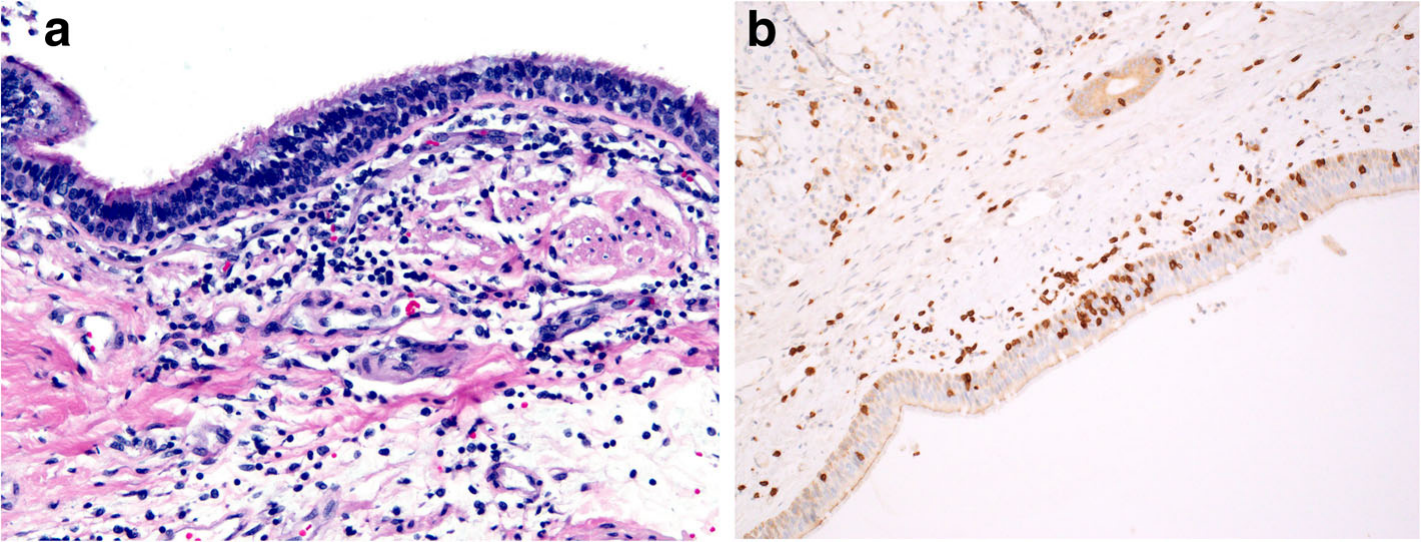
Airway inflammation in COVID-19: **a** Chronic inflammation composed mainly of lymphocytes, involving the bronchial mucosa (Hematoxylin & eosin, original magnification × 200). Note that blood vessels are free of thrombi; **b** CD3 demonstrates T-lymphocytes

Changes described in the airways (trachea, bronchi, and bronchioles) include chronic inflammation (Fig. 8a), and edema, resulting in thickening of the mucosa. The inflammatory infiltrate is mainly composed of CD3-positive T-lymphocytes (Fig. 8b).

The pathology described in other organs is far from specific, and it is unclear whether the changes described reflect viral infection or underlying/pre-existing conditions. Changes described in the liver include sinusoidal dilatation, macrovesicular steatosis, mild portal chronic inflammation, glycogenated nuclei, and mild lobular activity, accompanied clinically by an increase in liver enzymes [42].

Cardiac findings reported thus far have been non-specific and focal [27, 47]. They include “scattered individual myocyte necrosis and scattered lymphocytic infiltrate” in one case [69] and “lymphocytic myocarditis” in the right ventricle in another [47]. There is no pathologic evidence thus far that myocarditis is a common occurrence in COVID-19.

Bioinformatics analysis on single-cell transcriptome data showed that ACE-2 is predominantly expressed in the esophageal squamous epithelium and absorptive epithelium of the ileum and colon, suggesting that the virus may have the ability to infect and replicate in the gastrointestinal tract [70]. Autopsy of one case with COVID-19 showed segmental dilatation and stenosis of the small intestine, and a second case showed degeneration, necrosis, and shedding of enterocytes. Occasional lymphocytic infiltration of esophageal squamous epithelium was seen in some cases. Abundant lymphoplasmacytic infiltration of lamina propria of stomach, ileum, and rectum was also noted [71]. Viral nucleocapsid immunostaining showed presence of virus in the cytoplasm of gastric, duodenal, and rectal epithelium. As in other organs, it remains to be established whether these findings are related to viral damage or pre-existing pathology [71].

Reported renal findings include diffuse proximal tubule injury, tubular necrosis, loss of brush border, erythrocyte stagnation in the lumens of glomerular peritubular capillaries, and vacuolar degeneration in advanced cases [27]. Transmission electron microscopy has demonstrated viral particles in the cytoplasm of proximal tubular epithelial cells [27, 72].

Unlike coronavirus infection of pregnant women caused by SARS and MERS, COVID-19 during pregnancy has not yet been shown to impact maternal mortality. Currently, there is no evidence suggesting that SARS-CoV-2 can lead to intrauterine or transplacental transmission [73].

## Investigations

SARS-CoV-2 predominantly has a respiratory tissue tropism, although the virus can affect any tissue having ACE-2 receptors such as gastrointestinal, cardiovascular, and other tissues. In early infection, the virus is restricted to respiratory tissues, so the investigations to date are directed towards isolation of the viral nucleic acid from the saliva, sputum, nasopharyngeal secretion, and lower respiratory tract specimens. Of note, even after treatment and cure, the virus tends to be present in the stool for a prolonged period of time [29].

### Screening immunoassay for COVID-19

Immunoassays detect antigen-antibody reaction either by using monoclonal antibodies against the viral antigens or clonal viral antigens to identify antiviral IgA, IgG, and IgM antibodies. But they are less beneficial during the beginning of symptoms because the antibodies develop slowly over days to weeks before the titers become significant. Rapid antigen tests by immunoassay are being developed and their approval is still pending. Yet, they have the potential to become diagnosis of choice in the future because of their simplicity and rapidity [74].

### Specific molecular confirmation of COVID-19

A history of exposure, relevant systemic manifestations, and radiologic features of pneumonia make the diagnosis of COVID-19 more likely. But definitive diagnosis can only be achieved by a nucleic acid amplification test (NAAT) such as reverse transcription-polymerase chain reaction (RT-PCR) of the respiratory secretions [75]. As soon as the viral genome is sequenced, primers can be produced. Internationally, many laboratories use real time PCR assays by targeting two target gene sequences: E and RdRP. The assay devised by Center for Disease Control and Prevention (CDC) targets three different target sequences; NS3 (nucleotide sequence common among various SARS-like corona viruses), and N1 and N2 (specific for SARS-CoV-2). The NS3 assay over-diagnoses cases due to high false positivity rate. Then these false positive cases are eliminated by the specific assays targeting N1 and N2. Cycle threshold of less than 40 is considered positive (https://www.cdc.gov/coronavirus/2019-nCoV/lab/index.html). RNA extraction should be performed in a biosafety cabinet in a BSL-2 or equivalent facility. Heat treatment of samples before RNA extraction is discouraged, because RNA is prone to degrade swiftly. Although the lower respiratory tract secretion obtained by BAL procedure is the ideal specimen for testing as it has the highest viral load, sputum, and throat swab specimens are easier to procure and more practical for testing [76]. The rate of positivity of the throat swab specimen in early cases is around 60%, warranting caution to be applied in negative cases [77]. In COVID-19, viral load reaches its peak during the first week of disease. So, RT-PCR has a good detection rate during this period. The second week shows a gradual decline of viral load, yet is in the detectable range. Some asymptomatic cases may show a positive result. A proportion of recovered patients are virus carriers and may show positive test results [78]. RT-PCR is a sensitive test, yet it may show a negative result in a patient with a high degree of clinical suspicion. Two consecutive negative test results are needed for hospital discharge or discontinuation of quarantine [79]. Test accuracy is dependent upon various factors involving sample collection, the effectiveness of assays, and subjective ability of testing professionals. Nasopharyngeal swab is the practical choice for RT-PCR testing. It has to be performed with caution with proper insertion into nasopharyngeal space, otherwise, false negative rate will increase (https://www.cdc.gov/coronavirus/2019-ncov/lab/lab-biosafety-guidelines.html). RNA viruses generally have low fidelity polymerase enzyme resulting in the generation of high genetic diversity. Due to short genome length, one can use ‘genome walking’ in which primer pairs are designed for known nucleotide sequences and use them to determine the sequence of unknown genetic region in between them [80]. However, this method is time-consuming and has to be frequently reoptimized. These problems can be addressed by using next generation sequencing, by means of which a mammoth amount of sequences from a single sample can be generated. It also can detect and characterize a novel viral genome sequence without a need for specific primers [81]. Viral heterogeneity can be detected effectively by this method. Thus, it plays an important role in understanding the viral evolution [82]. Various techniques described above are illustrated in Table 3.

**Table 3:** Comparison between various diagnostic tests for COVID-19

Characteristics	Reverse transcriptase-polymerase chain reaction	Next-generation sequencing	Reverse transcription loop-mediated isothermal amplification technique	CRISPR techniques (DETECTR technique using CRISPR-Cas12)	Viral antigens detection	Antibodies detection
**Method**	Reverse transcription of RNA into cDNA strands, followed by amplification of specific regions of the cDNA	Reverse transcription of RNA into cDNA strands, construction of NGS library after amplifying full length genes, and sequencing analysis	Simultaneous reverse transcription and isothermal amplification at 37 degrees Celsius using a set of highly specific primers involved in annealing and synthesizing new strands, followed by appreciable color change to the naked eye that determines positivity	Simultaneous reverse transcription and isothermal amplification using RT-LAMPcas-12 detection of predefined coronavirus sequences, and cleavage of a reporter molecule confirms detection of the virus	Monoclonal antibodies detect viral antigens by immunoassay directly from the clinical specimens	Uses clonal viral antigens to detect antibodies (IgA, IgM, and IgG) to SARS–CoV-2 from clinical specimens (such as blood or saliva)
**Turn-around Time**	6–24 h	24 h	< 2 h	< 2 h	< 2 h	< 2 h
**Significance**	Highly specific and the test of choice	Comparison between various strains involved in the evolution of this illness, useful in research and vaccine development	Rapid, accurate, and relatively simple test with high specificity	Rapid, accurate, and relatively simple with high specificity	Rapid, simple, and potential future rapid test of choice	Rapid, simple, and can detect past infection or immunity from the infection (screening test)
**Drawback**	Complex technique, requires specialized laboratory and trained personnels	Complex technique and requires more time	Not quantitative	Not quantitative	Monoclonal antibody development in the laboratory is a time consuming and complex process	Antibodies become significant days to weeks after development of symptoms; not suitable for acute disease and disease confirmation

Other emerging diagnostic modalities are also focus of current research, particularly the tests having ‘point of care’ potential. One such example is the assays utilizing CRISPR (Clustered Regularly Interspersed Short Palindromic Repeat). They have dual advantage of being rapid as well as accurate. This technology can be used in resource poor countries, at the airports because of its simplicity, rapidity, portability, and low cost. DETECTR assay uses method of simultaneous reverse transcription and isothermal amplification using loop mediated amplification (RT-LAMP) [83]. This amplification procedure can be done at 37 degree Celsius, so there is no need of specialized thermal cyclers. It uses paper strip based detection making it very convenient. Other diagnostic modalities which are in the pipeline include lateral flow assays and microfluidic devices.

Recently four diagnostic tests have been granted emergency use authorization (EUA) by Food and Drug Administration namely 1) Modified CDC assay, 2) DiaSorin molecular simplexa, 3) GenMark, and 4) Hologicassay. These are basically modified versions of NAATs. Modified CDC uses same primers and probeset devised by CDC for RT-PCR, with an exception of N3. This improves the rapidity of the procedure. DiaSorin molecular simplexa targets the genes S and ORF1ab by differentiating with FAM and JOE fluorescent probes. RNA internal control (Q670 probe) is used to detect the failure of RT-PCR procedure. The analysis is done by LIAISON MDX studio software. Test result is considered positive if either of the two target genes can be detected. GenMarkePlex SARS-CoV-2 EUA panel uses a kit that amplifies N gene and detection is done by ePlex instrument. Hologic Panther Fusion® SARS-CoV-2 EUA is carried out using the manufacturer’s protocol using Panther Fusion Capture Reagent-S (wFCR-S). Magnetic field is used to separate the hybridized nucleic acid. ORF1ab gene is the target of this assay. Two conserved regions of this gene are amplified and detected in a fluorescent channel. Detection of only one of the two targets is considered positive [84].

### Viral detection by immunohistochemical and in situ hybridization asssays

SARS-CoV-2 viral protein can be detected in the tissue specimens using virus-specific antibodies and targeting viral RNA by in situ hybridization techniques [56, 59, 85–87]. Researchers have demonstrated in situ expression of SARS-CoV-2 in the airways and lung in the deceased COVID-19 individuals. In an autopsy study of seven cases with confirmed SARS-CoV-2 infection, the expression pattern of a rabbit polyclonal antibody against SARS nucleocapsid protein were correlated with clinical profiles. Of the five patients with acute-phase DAD (≤7 days from the onset of respiratory failure), SARS-CoV-2 was detected in the alveolar pneumocytes and ciliated airway epithelium in all 5 cases, and in the upper airway epithelium in 2 cases. In two patients with organizing DAD (> 14 days from onset of respiratory failure), virus was neither detected in the lungs nor the airways. No endothelial cell staining was observed. These observations suggest that SARS-CoV-2 infection of epithelial cells in the lungs and airways in patients with COVID-19 and respiratory failure can be detected during the acute phase of lung injury and is absent in the organizing phase [56]. In another study on the endomyocardial biopsies of 104 patients with suspected myocarditis or unexplained heart failure were analyzed by histology, immunohistochemistry, and detection of SARS-CoV-2 genomes by RT-PCR. Five of One hundred four biopsies were confirmed with SARS-CoV-2 infected by RT-PCR assay. Histopathology of the myocardium showed permeation into the vessel wall leading to small arterial obliteration and damage. They have speculated that myocardial injury and ischemia may play a role thus explaining the ubiquitous troponin increase in these patients. Furthermore, viral detection in the endomyocardial biopsy may be a potential therapeutic target of COVID-19 [59].

Another histopathologic, immunohistochemical, and electron microscopic study on the skin biopsies of 7 paediatric patients presenting with chilblains during the COVID-19 pandemic have shown interesting dermatopathologic features. Variable degrees of lymphocytic vasculitis, with endothelial swelling, damage, inflammation, fibrinoid necrosis, and thrombosis were identified. Purpura, superficial and deep perivascular lymphocytic inflammation with perieccrine accentuation, edema, and mild vacuolar interface dermoepidermal damage was seen. Interestingly, cytoplasmic granular positivity for SARS-CoV-2 spike protein was noted in the endothelial cells of the capillary and post-capillary venules of the upper dermis and in the epithelial cells of the secretory portion of eccrine units. Also, coronavirus particles were detected in the cytoplasm of the endothelial cells on electron microscopy [86]. The presence of viral particles in the endothelium and the morphologic evidence of endothelial damage, support a relationship between the clinical lesions and SARS-CoV-2 infection. The authors have proposed virus-induced endothelial damage may be the mechanism in the pathogenesis of COVID-19 chilblains and possibly also in a group of severely affected patients with features of widespread microangiopathy. This observation has also been supported by another study [87].

### Nonspecific associated investigations

Nonspecific laboratory findings include leukocytosis with lymphopenia [17]. Though lymphopenia is seen, the lymphocytes are hyperactivated as suggested by their double positivity for HLA-DR and CD38 and presence of CD8 + T cells with high concentration of cytotoxic proteins [38]. There is an elevation in serum concentration of liver enzymes, muscle enzymes, myoglobin, lactatedehydrogenase, and acute phase reactants. Critical cases also show increased concentration of procalcitonin, D-dimers, and severe lymphopenia. Exploring the cytokine dynamics in one study showed increased plasma concentration of interleukin (IL)1B, IL1 receptor antagonist, IL7, IL8, IL9, IL10, basic fibroblast growth factor, granulocyte colony-stimulating factor, granulocyte-macrophage colony-stimulating factor, interferon γ, IP10, MCP1, MIP1A, MIP1B, platelet-derived growth factor, tumor necrosis factor, and vascular endothelial growth factor were identified in the patients compared to healthy adults [34]. Also, a persistent elevation of IL-6 has been observed in COVID-19 patients, and tocilizumab appears to be an effective therapeutic option in these patients who are at a risk of developing cytokine storms [88]. In advanced cases with multiorgan dysfunction/failure and cytokine storm, there is an overwhelmingly high concentration of the cytokine mentioned above (s) playing a significant role in the disease pathogenesis and is responsible for the significant catastrophe [89].

## Issues on laboratory safety

Cytology laboratories deal with handling of body fluid including respiratory secretion necessitating stringent precautionary measures while handling suspected COVID-19 specimens. General measures of social distancing should be followed. Working in shifts and brief meal breaks should be promoted. In addition to respiratory samples and peripheral blood, the virus can be present in the stool and urine. Virus can also be present in asymptomatic, non-diagnosed, and convalescent cases. So, universal standard precaution has to be followed strictly [90]. Rapid on-site examination of the cytology specimen during fine needle aspiration (FNA) procedure should be avoided. In case of absolute necessity, appropriate personal protective equipments (PPE) such as goggles or face shields and filter respirator N95 or higher level should be used. FNA procedures should be avoided unless it is urgent. Air drying or heat drying should be done under class II biosafety cabinets. Special precautions are required in the procedures that can generate aerosols including during sample collection and cytopreparation, particularly during mixing, vortexing, pipetting, and aliquoting. These procedures should also be done under class II biosafety cabinets. Agitating the smear should be avoided. As the virus is inhibited by formalin and alcohol solutions, over 70% alcohol concentration, most cytology specimens, and cell blocks are considered safe. However, the use of gloves while handling slides is encouraged. Access to the laboratory should be limited. Laboratory personnel should be appropriately trained regarding the use of PPEs. Frequent hand washing for at least 20 s is encouraged. Decontamination of all work surfaces is recommended. Identification and segregation of contaminated material should be adequately carried out. If decontamination cannot be carried out in the laboratory area, contaminated wastes must be carefully packaged in a leak-proof container and transferred to a facility capable of handling the decontamination process (https://www.cdc.gov/coronavirus/2019-ncov/lab/lab-biosafety-guidelines.html).

An optimized autopsy protocol for COVID-19 cases have been devised by various institutions and regulatory bodies [27, 91]. Usually, 2 h prior to the procedure, 4% phosphate-buffered formalin was instilled into the mouth, nostrils, and pharynx. The airflow (> 6 air changes per hour of total room volume) should be adequate in the autopsy suite and the autopsy should be performed at the conditions similar to recommendations for autopsies of suspected Creutzfeld-Jakob disease (i.e. hazmat suits, boots, goggles, FFP2/3 masks); anin-corpore technique analogous to that used in forensic institutions may be applicable.

An advanced biomarker-driven proteomics and genomics platform to identify the protein and nucleic acid machinery of the virus and its interaction with human cell is essential to diagnose these cases. Performing COVID-19 genome sequencing will enable us to monitor current outbreaks in real-time, especially in highly-populous urban areas across the globe, and understand how the virus continues to mutate (epitope drift and shift) and spread in our diverse communities. This could notably help us understand a number of issues, including how many different infection clusters there are, and how many new clusters develop overtime, for each cluster, trace the origin of the virus, for each cluster, trace the speed of the spread, to identify specific communities in which the virus is spreading more rapidly, to track the speed of the overall community spread, to identify how many different sources of infections are currently present, to see the similarities and differences between virus strains circulating, and their comparison among various geographic location, to identify which mutations in the virus causing most severe symptoms and the relationship between various mutations and their clinical phenotypes. Additionally, affected individual’s cardiovascular and coagulation status need to be taken into consideration while determining the severity of the disease/infection. This would eventually assist in the development of more efficient mRNA based vaccines, or oligonucleotide-based therapies.

In conclusion, the main pathologic manifestation of COVID-19 based on initial reports is DAD accompanied by thrombotic complications, and the main site of pathologic abnormality is the lungs. An enhanced knowledge of the pathogenesis is critical as new therapeutics and vaccine trials have started. A precise understanding of the underlying basis of this disease and its spread and natural history may aid in the selection of new therapies. Further research into the pathology of this disease is sorely needed.

## Data Availability

This is a review article and does not contain any data, therefore, deemed not applicable.

## Abbreviations

COVID-19: Coronavirus disease-2019
SARS-CoV-2: Severe Acute Respiratory Syndrome Coronavirus-2
ACE-2: Angiotensin-converting enzyme-2
ARDS: Acute respiratory distress syndrome
MERS: Middle East Respiratory Syndrome
GISAID: Global Initiative on Sharing All Influenza Data
NCBI: National Center for Biotechnology Information
UTR: Untranslated regions
ORF: Open Read Frame
RdRP: RNA-dependent RNA polymerase
WHO: World Health Organization
DAD: Diffuse alveolar damage
RT-PCR: Reverse transcription-polymerase chain reaction
NAAT: Nucleic acid amplification test
CDC: Center for Disease Control and Prevention
BSL-2: Biosafety level-2
CRISPR: Clustered Regularly Interspersed Short Palindromic Repeat
RT-LAMP: Reverse transcription and isothermal amplification using loop mediated amplification
EUA: Emergency use authorization
wFCR-S: Panther Fusion Capture Reagent-S
FNA: Fine needle aspiration
PPE: Personal protective equipments
